# Building The STEM Village: an interview on fostering LGBTQ+ visibility and community

**DOI:** 10.1038/s42003-022-04181-3

**Published:** 2022-11-18

## Abstract

The STEM Village is an international effort dedicated to improving the visibility of the LGBTQ+ STEM community. As part of our own celebration of LGBTQIA+ STEM Day, we spoke to members of The STEM Village leadership team about their programming and ways to get involved.

**Figure:**
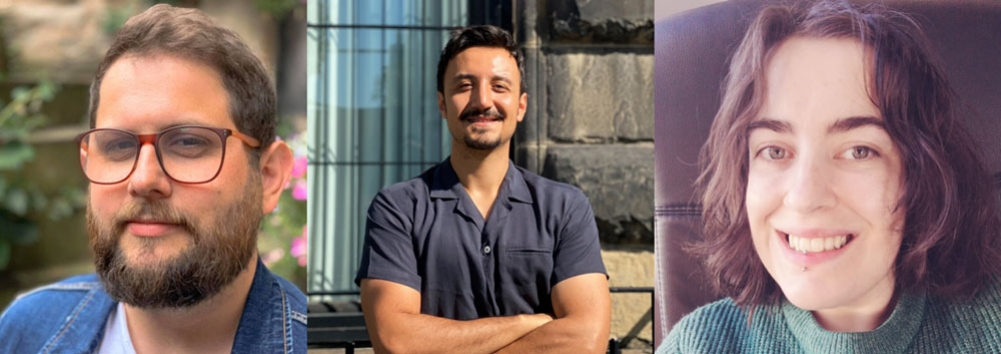
Members of The STEM Village leadership team. Pictured from left to right: Dr. Matthew Sinton (he/him), Dr. Mehmet Kurt (he/him), and Kiri Thornalley (they/them).

To start off, could you each please tell us a little bit about your background, as well as any current research projects?

**Matthew Sinton (MS):** My academic path has been fairly circuitous, and I took time away from academia after my undergraduate degree to try out alternative careers. However, I always missed research, and eventually made the decision to make my way back to academia. My Ph.D. focused on understanding the interplay between carbon metabolism and epigenetics during the pathogenesis of non-alcoholic fatty liver disease, using a novel human stem cell-based model. I am absolutely in love with metabolism and following on from my Ph.D. decided that I wanted to understand more about immunometabolism and energy balance during infection and disease. I recently developed a project, with the help of my husband, using a parasite (*Trypanosoma brucei*) to understand infection-induced weight loss and the role of sex in this weight loss. It’s been super interesting and has thrown open a million more questions, which is very exciting.

**Mehmet Kurt (MK):** I’m an Assistant Professor of Mechanical Engineering at the University of Washington. I have received my Ph.D. in Mechanical Science and Engineering from the University of Illinois at Urbana-Champaign and completed my doctoral studies in bioengineering at Stanford University. I made a drastic transition from studying nonlinear dynamics during my Ph.D. to investigating brain biomechanics through neuroimaging during my postdoctoral studies. My current research program represents a combination of these two concepts. My lab, Kurtlab, aims to merge advanced neuroimaging tools, multi-physics brain modeling, and machine learning for the in vivo, subject-specific investigation of brain mechanics.

**Kiri Thornalley (KT):** Currently I’m a Ph.D. student in Pharmacy and Biomedical Sciences, at the University of Strathclyde, Glasgow. In order to develop methods which could be used in lieu of animal-based pre-clinical testing for novel therapeutics, my research uses particle metrology, computational fluid dynamics and microfluidics to study the composition of the biomolecular corona around polymer nanoparticles, the influence the proteins contained within the corona have on nanoparticle biological fate and how these factors are impacted by fluid shear in vivo.

How would you describe The STEM Village to someone who has not heard of it before?

**MS:** The idea of The STEM Village is to boost visibility for LGBTQ+ people in STEM, and to dismantle the heteronormative idea of who can be involved in STEM or have a career in STEM. We also want people to have the opportunity to network so that they have the opportunity to form new collaborations and support networks.

**KT:** For me, The STEM Village are my found family and I’d go as far as to include LGBTSTEM and #LGBTSTEM Twitter here too. They are the people who have kept me in STEM, despite me not always feeling like I fit in here (I’m non-binary and have lived experience of severe and enduring mental illness), because they would care if I left.

It’s often said it takes a village to raise a child. I’d say it takes a village to raise a researcher; a Ph.D. isn’t something you do on your own. The original aim of The STEM Village was to create a community of LGBTQ+ researchers based in Scotland, then the pandemic happened so everything became online and at that time we saw very little point of limiting it to just researchers based here in Scotland.

**MK:** It really is a platform that aims to increase LGBTQ+ visibility in STEM by providing the necessary space for networking and outreach. The STEM workplace is often not a place where LGBTQ+ people feel able to be out and visible. We hope that efforts from initiatives such as The STEM Village will change that eventually. I have been actively involved with The STEM Village since 2020, including organizing a virtual conference, workshops and events.

Matthew: What inspired you to start The STEM Village?

**MS:** The STEM Village was inspired by the groups Pride in STEM and LGBTQ+ STEM who were already doing fantastic work and organizing some brilliant events. At that time, the events were more focused south of the Scottish border and I realized that there wasn’t really a similar initiative in Scotland. The idea at the time was to start a network in Scotland, to boost the visibility of LGBTQ+ in STEM and to create a Scottish network. We raised funding for the event, and it was all set to go ahead, and then the pandemic hit. As many others did, we pivoted to an online event, which was actually one of the most worthwhile things I’ve done. We were able to host LGBTQ+ people from across the world, over a 12-hour period, to talk about their work and their experiences. We even hosted a drag in STEM panel, where scientists who are also drag performers shared their experiences. The event was also supported by the leader of the Scottish government, Nicola Sturgeon, and it was incredible knowing that the leader of our nation was willing to throw her support behind our community. I think the highlight of this event was a session that was a conversation between a mentor and mentee who have been friends for a long time, and they discussed their experiences of being queer in STEM at quite different times in history. It was beautiful to hear their stories and their experiences.

Mehmet and Kiri: How did you first get involved in The STEM Village?

**MK:** I remember seeing a tweet from The STEM Village on my Twitter feed and reaching out to Matthew at the beginning of the pandemic (thank you Twitter algorithm!). He kindly invited me to give a virtual talk in one of The STEM Village events and then I started to be actively involved. We collaborated on organizing the global LGBTQ+ STEM conference in August 2020. It was really comforting to be a part of this amazing group during the peak of the pandemic.

**KT:** I’m almost certain that I knew Matthew via Twitter before I arrived in Glasgow late 2019. Twitter direct messages suggest that I’d just wanted to get involved with the talk series he was running online, then it very much blossomed from there. A few months later, I was then involved with the virtual symposium in August 2020.

Mehmet: One complementary program to The STEM Village is the PRIDE mentorship scheme. Could you please talk a little bit about the purpose of this program and its inception?

**MK:** Sure! The main objective of Peer Review for Inclusion, Diversity, and Equity (PRIDE) program is to create a database of reviewers who volunteer to review fellowship applications of LGBTQ+ and other underrepresented minority students in STEM. Our primary objective is to foster a sense of community among LGBTQ+ researchers in STEM and we hope that organic, long-lasting mentor/mentee relationships will form through our program. Since its launch, we helped form several mentor/mentee relationships and I would love to grow this program! If any of the readers would like to volunteer for or utilize the PRIDE program, please visit our website (www.kurtlab.com/pride) and send us an email at peerreviewpride@gmail.com

On another note, our current plans are to expand the reach of PRIDE and start a pilot LGBTQ+ mentorship program. We will pair LGBTQ+ students in higher education who wish to be mentees with industry or academia professionals in the fields of their research interests. Professionals will work with students in an 8-week program where students will complete a research project and receive career advice. We are currently working on this, so stay tuned!

What has been your favorite STEM Village initiative so far?

**KT:** The STEM Village Virtual Symposium! Incredibly stressful from my end, but also so much fun. I know from other attendees this looked really slick, simultaneously broadcasting on Zoom and YouTube – so that those for whom it wasn’t safe to be out, could still attend. I was running around like a headless chicken behind the scenes, spreadsheets everywhere so I knew which talks we’d had, which ones were recorded, which ones required editing before being uploaded to YouTube as standalone videos as presenters hadn’t consented for their talks to be recorded. Two years later and I still cannot believe we only lost one speaker due to internet issues and somehow got a recorded talk from Nicola Sturgeon (the First Minister of Scotland).

**MK:** Definitely the global, virtual LGBTQ+ STEM Conference in August 2020! The conference included registrations from individuals in countries where it is illegal/dangerous to be openly LGBTQ+, which was really moving. The event itself was attended by approximately 700 individuals, who would otherwise have been unable to participate, due to geographical, societal, political or legal restrictions. The way the community came together during this conference was a cathartic experience: Featured talks from notable LGBTQ+ scientists, discussions about the unique challenges faced by the LGBTQ+ STEM community… It was an amazing experience!

What advice would you give to trainees or early-career researchers who might want to start similar programs?

**KT:** If you have a particular protected characteristic and you’re lonely because you feel like you’re the only one in your department, there will absolutely be other people like you who also feel lonely and isolated in their respective departments. Take to social media (e.g., Twitter) and you will find your people.

**MS:** To anyone who is thinking about starting a similar initiative, I would say to absolutely go for it. Reach out to like-minded people, whether by email or on social media, and figure out what you want your group to achieve, whether it’s a local campus-based initiative or something broader. People are really receptive to these kinds of initiatives and keen to get involved and offer support wherever they can. We’ve been supported by the British Society for Immunology and the Scottish Universities Life Sciences Alliance, as well as the Universities of Edinburgh, Glasgow, and Strathclyde, and I know that there are many organizations who are happy to support these types of events in any way that they can.

**MK:** I would definitely recommend first reaching out to the local LGBTQ+ organizations in their institutions for collaboration (e.g., oSTEM chapters). I think community outreach and effective use of social media are also important for success — that’s how I discovered The STEM Village to begin with!

What are ways to get involved with The STEM Village (for anyone inspired by reading this Q&A)?

**KT:** I’m going to defer to Matthew for this one!

**MS:** If people would like to find out more about The STEM Village, they can read about us on our website (www.thestemvillage.com), check out videos on our YouTube channel, follow us on Twitter (@thestemvillage) or email us at info@thestemvillage.com. We’ve been pretty quiet recently due to other commitments but we’d still love to keep connecting with the community!

**MK:** Just reach out to us! If there are any events/workshops you would like to organize, we would love to collaborate with you!

*This interview was conducted by Senior Editor George Inglis*.

